# Concise Review: Mesenchymal Stem Cells Ameliorate Tissue Injury via Secretion of Tumor Necrosis Factor-*α* Stimulated Protein/Gene 6

**DOI:** 10.1155/2014/761091

**Published:** 2014-12-15

**Authors:** Zhigang He, Jie Hua, Zhenshun Song

**Affiliations:** Department of General Surgery, Shanghai Tenth People's Hospital, Tongji University School of Medicine, 301 Yanchang Middle Road, Shanghai 200072, China

## Abstract

Numerous reports have described therapeutic benefits in various disease models after administration of the adult stem/progenitor cells from bone marrow or other tissues referred to as mesenchymal stem cells/multipotent mesenchymal stromal cells (MSCs). They all showed that one of the important effects of MSCs is to act against excessive inflammatory responses and repair the damaged tissues. The therapeutic benefits of MSCs were initially interpreted by their migration, engraftment, and differentiation into target tissues. However, remarkable anatomical structural repairs and functional improvements were increasingly observed with a small number of or even no MSCs in the injured tissues. This suggests that most beneficial effects are largely due to paracrine secretions or cell-to-cell contacts that have multiple effects involving modulation of inflammatory and immune responses. Currently, the therapeutic benefits of MSCs are in part explained by the cells being activated by signals from injured tissues to express an anti-inflammatory protein, tumor-necrosis-factor-*α*-induced protein 6. This important mechanism of action has attracted increasing attention, and therefore we conducted this review to summarize the latest research.

## 1. Introduction

Inflammatory response is a common and important basic pathological process, which is generally recognized as an essential but complex defense mechanism in tissue injury. In fact, there is also an increasing realization that excessive or nonresolving inflammation makes a major contribution to the damage wrought by diseases [1–7]. Traditional treatments for tissue injury are often limited in number and below expectation in their therapeutic effects. Therefore, it is necessary to explore more effective treatments and their modes of action. One concern is that numerous studies have demonstrated therapeutic benefits in various diseases associated with inflammation and injury after administration of the adult stem/progenitor cells referred to initially as colony forming units-fibroblastic, then as mesenchymal stem cells or multipotent mesenchymal stromal cells (MSCs) [8–15]. MSCs are characterized by the ability to self-renew and undergo differentiation into mesenchymal lineage cell types including bone, cartilage, adipose tissue, and muscle and have been confirmed to play a role in immune regulation, cell-growth adjustment, and tissue repair [9, 16–20]. The therapeutic benefits of MSCs were initially interpreted by their migration, engraftment, and differentiation into target tissues. However, remarkable anatomical structural repairs and functional improvements were increasingly observed, with a small number or even no MSCs in the injured tissues. This suggests that most of the beneficial effects are largely due to paracrine secretions or cell-to-cell contacts that have multiple effects involving modulation of inflammatory and immune responses [21–23]. The inflammatory microenvironment can play a specific regulatory role in the paracrine activity of MSCs and these secreted mediators also have a role in the damaged target tissues or organs [24–27]. Many recent studies have suggested that the therapeutic benefits of MSCs are in part explained by the cells being activated by signals from injured tissues to express an anti-inflammatory protein, tumor necrosis factor- (TNF-) *α*-induced protein (TNAIP)6, or TNF-*α*-stimulated gene- (TSG-) 6 [20, 28–31].

## 2. Correlation between MSCs and Inflammation or Tissue Injury

Friedenstein et al. reported firstly in 1968 that bone marrow contains a large number of stem cells, and Caplan et al. named them MSCs [16, 25, 32, 33]. Accumulating evidence shows that MSCs may be the most promising choice for research in tissue engineering and regenerative medicine because of their immunosuppressive and anti-inflammatory properties [34, 35]. Although MSCs are involved in phase II clinical trials, many problems remain to be resolved, such as their long-term effects and precise mechanisms of action. MSCs have been shown in graft versus host reaction, osteogenesis imperfecta, Crohn's disease, and spinal cord injury [36–39] with remarkable therapeutic effects. These therapeutic effects do not solely rely on the engraftment or differentiation of MSCs because only a small number of MSCs were observed in the injured tissues, which suggests that MSCs repair the tissues largely through their paracrine effects. By reacting with the target tissue, MSCs will secrete a variety of therapeutic substances including cytokines, growth factors, cell-signaling molecules, and exosome [25, 33, 40–48].

**Figure 1:**
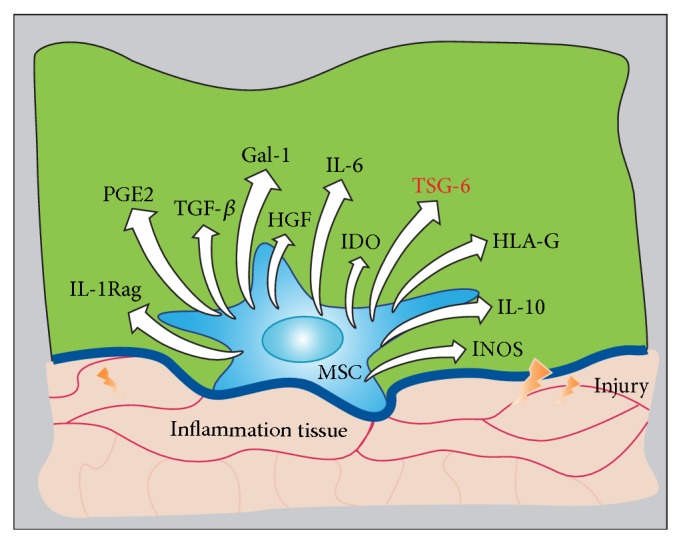
MSCs interaction with immune cells or inflammatory cells during inflammation results in production of many cytokines that moderate the inflammatory environment. MSCs have been confirmed to affect immune regulation and tissue repair. In the inflammatory environment of injured tissue, MSC interactions with T cells, macrophages, and dendritic cells result in inhibition of inflammation. Many factors have been identified as contributing to this response, especially TSG-6.

In the microenvironment of inflammation, MSCs can interact with immune cells and produce at least 11 soluble cytokines: TSG-6, hepatocyte growth factor, transforming growth factor (TGF)-*β*, prostaglandin (PG) E2, interleukin- (IL-) 6, IL-10, and IL-1 receptor antagonist, inducible NO synthase, indoleamine 2,3-dioxygenase, galectin-1, and human leucocyte antigen (HLA)-G [20] (Figure 1). Thus, it can be concluded that MSCs are capable of producing sufficient cytokines to suppress inflammation and injury. TSG-6 may, however, play a key role in many beneficial effects of MSCs.

## 3. TSG-6: A Multifunctional Protein Associated with Inflammatory Injuries

**Figure 2:**
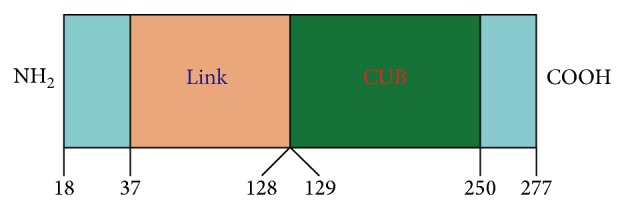
Structure of human TSG-6. TSG-6 is a ~35 kDa secreted protein composed mainly of contiguous link and CUB modules. On the basis of our structural and modelling studies, these can be defined as residues 37–128 and 129–250 in the preprotein.

TSG-6 is a new type of gene that maps human chromosome 2q23.3. It was originally identified as a cDNA derived from TNF-*α*-treated human fibroblasts, and its corresponding expressive protein (TNAIP6) is mainly composed of a contiguous link module and complement subcomponents C1r/C1s-Uegf-BMP-1 (CUB) module [28, 49] (Figure 2). TSG-6 is capable of combining with hyaluronic acid (HA), chondroitin sulfate, protein polysaccharide or aggrecan G1 chain, inter-*α*-inhibitor (I*α*I), and remodeling extracellular matrix (ECM) [50–54]. There is no set distribution of TSG-6 in humans or mammals. It has been confirmed that TSG-6 is a protective inflammatory gene that is present in different cell types [28, 29, 55]. The promoter sequence of TSG-6 contains binding sites for activator protein and nuclear factor interleukins, which can be activated by proinflammatory factors [56]. It is also becoming clear that TSG-6 is produced in inflammatory processes such as rheumatoid arthritis and inflammation-like processes such as ovulation and cervical ripening, where its expression is probably induced by growth factors (e.g., TGF*β*), epidermal growth factor, fibroblast growth factor, and PGE2 [31, 57–59]. ECM remodeling is a key feature of most, if not all, the known sites of TSG-6 expression, indicating that it might participate in this process. By contrast, both the protein sequences encoded by TSG-6 and HA-binding protein (e.g., CD44, cartilage link protein) contain the highly conservative area binding with HA, and homology is as high as 40% [60–62]. CD44 is the major cell surface receptor for HA, and CD44-HA interactions contribute to leukocyte rolling during inflammation. Proinflammatory cytokines increase HA expression on the vascular endothelium and induce the HA-binding capacity of CD44^+^ leukocytes, thereby promoting the contribution of HA-CD44 interactions to leukocyte migration. Lesley et al. have found that TSG-6 can integrate into HA and thus might mediate its anti-inflammatory effects by blocking this interaction [63–65]. In addition, it has been confirmed that TSG-6 suppresses inflammation also via inhibiting the vitality of I*α*I. I*α*I comprises two heavy chains and bikunin chains. Bikunin is associated with inhibition of I*α*I. However, TSG-6 can transfer the heavy chains from I*α*I to HA thus resulting in the release of activated bikunin so that it can achieve the above purpose [63].

## 4. MSCs Ameliorate Tissue Injury through Secreted TSG-6

### 4.1. Lung Injury

Lung injury is a common pathological process. It has been shown that application of MSCs in an experimental model of lung injury has a surprising therapeutic effect [66]. Danchuk et al. [67] have elucidated the role of human MSCs (hMSCs) in lung injury. The lungs of immunocompetent mice were exposed to lipopolysaccharide (LPS) and 4 h later 5 × 10^5^ hMSCs, or human lung fibroblasts (hLFs) as a control, were delivered by oropharyngeal aspiration. At 24 and 48 h after LPS, animals were euthanized by exsanguination and the lungs were processed for bronchoalveolar lavage, RNA extraction, histology, or lung wet/dry weight measurements, to assess inflammation and lung injury. Aggarwal and Pittenger [19] have found that hMSCs can significantly reduce the expression of proinflammatory cytokines, the number of neutrophils, and the extent of pulmonary edema, but hLFs do not. The anti-inflammatory effect may not rely on differentiation of hMSCs homing to sites of lung injury, because equivalent numbers of hMSCs given by intraperitoneal injection can also significantly suppress inflammatory cell accumulation in the lungs. Through using gene chip technology when hMSCs were injected into ALI mice, one of the most highly upregulated human genes identified in LPS-exposed lung encodes TSG-6. This experiment showed that the anti-inflammatory effect of hMSCs is largely abrogated after TSG-6 mRNA knockdown, but delivery of recombinant human TSG-6 (rhTSG-6) can achieve a similar effect to hMSCs. All these results suggest that TSG-6 mediates, in part, the anti-inflammatory effects of hMSCs.

In 2014, Foskett et al. [68] also demonstrated that MSCs, targeting early inflammation, can improve bleomycin-injured lungs by secreting TSG-6. Furthermore, TSG-6 also plays an important role in idiopathic pulmonary arterial hypertension and asthma, which demonstrates that TSG-6 may be an important mediator secreted by MSCs to improve pulmonary inflammatory injury [69, 70].

### 4.2. Acute Myocardial Infarction

Acute myocardial infarction (AMI) is a serious and life-threatening disease that arises from acute, prolonged coronary ischemia [71, 72]. A mouse model for AMI can be created through permanent ligation of the anterior descending coronary artery [73]. Using this model, Lee et al. [74] observed the therapeutic effects on AMI of intravenous infusion of MSCs (2 × 10^6^ cells/mouse). MSCs significantly reduced the early inflammatory response to permanent ligation of the anterior descending coronary artery and subsequently reduced infarct size. Significant improvement was also observed in the function of the left ventricle as assayed by echocardiography 3 weeks later. However, quantitative assays for human Alu sequences and human mRNA for GAPDH as a measure for live hMSCs indicated that only a small fraction of the infused hMSCs (400 ± 300, 1480 ± 530, and disappearance of the 2 × 10^6^ cells were present at 15 min, 24 h, and 48 h, resp.) were recovered from the heart after injection, and most were trapped in the lungs as microemboli. RNA was extracted from the lungs of the mice 10 h after infusion of the hMSCs and was assayed using human microarrays. The data indicated that the expression of over 50 human genes, such as SMAD6, CSF1, VCAM-1, and TSG-6, was upregulated. However, TSG-6 expression was 26–47 times higher than the basal level and was far higher than expression of the other human genes. TSG-6 was also expressed in other cell types apart from hMSCs when they were in the same environment. TSG-6 is a multipotent anti-inflammatory protein that is secreted by hMSCs, and it may exert vital therapeutic effects on AMI. In a further attempt to explain the beneficial effects of TSG-6 secreted by hMSCs, hMSCs with TSG-6 knockdown or rhTSG-6 was infused intravenously into the body of AMI model mice. The former had little or no effect in the AMI model but the latter had the same beneficial effect as hMSCs. How do we explain this phenomenon? By being activated to secrete TSG-6, MSCs acted at a distance to reduce injury to the heart.

### 4.3. Peritonitis and Peritoneal Injury

The application of MSCs in peritonitis and peritoneal injury is still being explored. Although the tissue repair function and immunoregulation of MSCs have been known for a long time, their exact mechanisms of action are still not clear. In 2011, the anti-inflammatory action of MSCs through secretion of TSG-6 was further demonstrated in a peritonitis model by Choi et al. [75]. Zymosan, a glucan prepared from the cell walls of yeast, can activate macrophages via Toll-like receptor (TLR) 2 and can be used to induce a mouse model of peritonitis. In a series of* in vitro* experiments, they demonstrated that TSG-6 interacts through the CD44 the murine resident RAW 264.7 macrophages to decrease zymosan/TLR2-mediated nuclear translocation of NF-*κ*B. After that, expression of proinflammatory cytokines is decreased and in turn reduces stimulation of MSCs. Therefore, the whole process becomes a degenerative feedback system and the endothelial cells or other cells can also amplify this feedback effect.

Wang et al. [76] demonstrated that intravenous administration of MSCs can also improve peritoneal injury in rats. However, the MSCs with siRNA knockdown of the TSG-6 gene had little or no effect in this model. Besides, conditioned medium from starved MSCs had a beneficial effect on peritoneal injury, and the content of TSG-6 increased 194 times compared to that in cells that had not been starved. Subsequent experiments confirmed that peritoneal adhesion can be improved by TSG-6 secreted through MSCs.

### 4.4. Brain Injury

Brain injury is a result of cerebral ischemia or trauma. Its recovery depends on the number of surviving neurons in the brain, especially the region impaired or infarcted. Watanabe et al. [77] have demonstrated that MSCs can ameliorate traumatic brain injury in mice. Secreted TSG-6 acts as a mediator and plays a vital role, because it decreases the number of neutrophils and expression of matrix metalloproteinase-9, which improves leakage from the blood-brain barrier. In 2013, Lin et al. [78] found that the amount of S-100B in ischemic brain injury is decreased by intravenous administration of MSCs and intracerebroventricular injection of rhTSG-6. This effect also relies to a large extent on the action of the secreted TSG-6.

### 4.5. Corneal Injury

Corneal injury may be the most promising field for therapy with MSCs. Intravenous administration of MSCs had surprising therapeutic effects in a model of sterile corneal injury. Oh et al. [79, 80] found that, among neutrophil infiltration, production of proinflammatory cytokines and development of opacity in the cornea were markedly decreased, even though few MSCs were present. In addition, intraperitoneal administration of MSCs was effective in suppressing inflammation and preventing corneal opacity. That is to say, differentiation of MSCs may not exert the main effects, but rather the paracrine function does. Quantitative assays of human mRNA for GAPDH also indicated that <10 cells were present in the corneas of rats at 1 and 3 days after intravenous or intraperitoneal administration of MSCs. In order to explain the beneficial effects of MSCs, rhTSG-6 and MSCs with siRNA knockdown of the TSG-6 gene were also applied to a model of corneal injury by brief exposure to alcohol. The latter were not effective but the former decreased excessive inflammation in the injured cornea. Therefore, systemically administered MSCs reduced inflammatory damage to the cornea, without engraftment, and the anti-inflammatory effects of the cells were probably explained by their secretion of TSG-6 [81].

## 5. Conclusion

**Figure 3:**
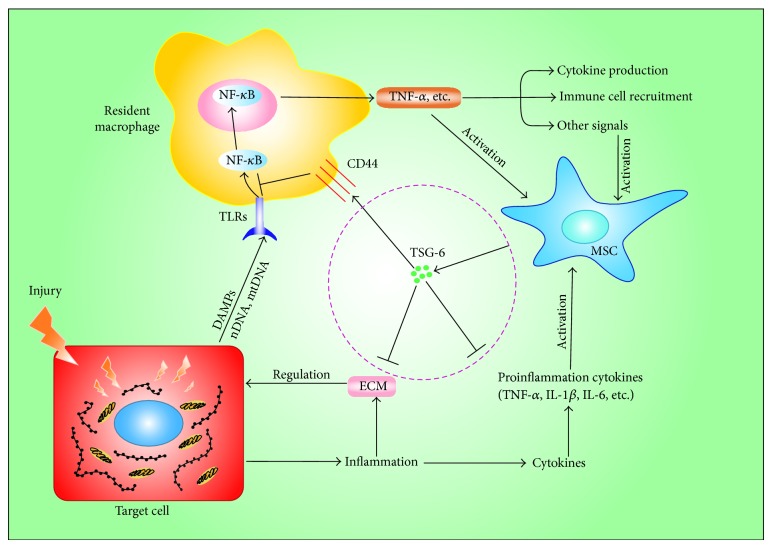
Anti-inflammatory action of MSCs is mediated mainly through TSG-6. Damage-associated molecular patterns (DAMPs), as a result of tissue injury, activated resident macrophages via Toll like receptors (TLRs) and nuclear factor-*κ*B (NF-*κ*B) to increase the expression of proinflammatory cytokines. In addition, the target cells could also secrete a number of proinflammatory cytokines when they were damaged. In turn, the production of cytokines and other signals would engage MSCs to secrete TSG-6, which negatively regulated TLRs/NF-*κ*B signaling through binding the CD44 receptors on the macrophages or suppress inflammation via extracellular matrix (ECM) reorganization.

In addition to the abovementioned conditions, MSCs can also achieve a satisfactory therapeutic effect in diabetes mellitus and skin injury, and this is also primarily reliant on the role of TSG-6 [82, 83]. Damage-associated molecular patterns as a result of tissue injury always contribute to a series of inflammatory and immune reactions through the pattern recognition receptors such as Toll-like receptors [84–86]. When MSCs are placed in the inflammatory microenvironment, they are activated by special signals from the target cells to increase the expression level of TSG-6 [87]. Furthermore, TSG-6 combines with HA or I*α*I and thus exerts its anti-inflammatory action (Figure 3). However, it is still not clear whether the MSCs that come from the umbilical cord, adipose tissue, and other tissues, apart from bone marrow, all possess similar therapeutic effects via secreted TSG-6. The action of TSG-6 also still needs further exploration and research. Deepening our understanding of the complicated mechanism through which MSCs exert their anti-inflammatory effects will yield more valuable information about MSCs and provide inflammatory diseases or tissue injury with new therapeutic options.
